# Benefits of a national network of drug information centres: RELIS

**DOI:** 10.1007/s00228-016-2129-7

**Published:** 2016-09-15

**Authors:** Jan Schjøtt

**Affiliations:** 1 Section of Clinical Pharmacology, Laboratory of Clinical Biochemistry, Haukeland University Hospital, PB 5021 Bergen, Norway; 2 Department of Clinical Science, Faculty of Medicine and Dentistry, University of Bergen, Bergen, Norway; 3 Regional Medicines Information and Pharmacovigilance Centre (RELIS Vest), Haukeland University Hospital, Bergen, Norway

RELIS is a Norwegian network of drug information centres providing drug information to health care professionals and patients in four health regions. The regional centres process adverse drug reaction reports (ADR reports) into the ADR database administered by the Norwegian Medical Agency. This includes provision of feedback about causality to the reporting health care professionals. The centres are associated with clinical pharmacology units in regional university hospitals, and pharmacists and physicians with expertise in searching and critical evaluation of literature constitute the staff in the centres. The aim of this letter is to highlight how the network model could be of international interest.

A central tool among our shared internet-based resources is the RELIS database with nearly 40,000 question-answer pairs. Health care professionals can access published cases from the database for free through a new home page (www.relis.no) launched in 2015 and sign up for regular newsletters distributed to 5100 subscribers. In the home page, the staffs also publish review articles on relevant themes.

In 2011, RELIS launched its internet-based service for pregnant and breastfeeding. Safe Mommy Medicine (www.tryggmammamedisin.no) provides exclusive answers about risks associated with drug treatment within two working days. At the end of June 2016, nearly 12,000 questions have been handled, and at the start of May 2016, we opened an additional telephone service. A similar internet-based service for general drug-related questions from patients and the public, Safe Medicine (www.tryggmedisin.no), opened in the autumn 2014 with nearly 1000 questions handled.

The network share exclusive e-mails based on a web question form available on the homepage for health care professionals. Thus, regardless of location, new questions submitted through the homepage are available to all RELIS staff. Furthermore, a RELIS intranet serve as a working-platform for documentation and communication within the network. The intranet contains an application centre where the staff has access to store and index new data or to retrieve old data. This includes access to not-published cases (e.g. copy of a previous answer, lack of public interest) in the RELIS database and the two databases for queries from the public. This provides the possibility of tracing questions (also by telephone or ordinary e-mails) from health care professionals and public. Staff members who are handling (answer in progress) or have handled a question can be contacted in case of a related question or need of particular competence [1].

A Norwegian academic detailing program project which involves knowledge-based update visits to general practitioners (GPs) started 2015 by the Department of Clinical Pharmacology, St. Olavs Hospital, Trondheim. Two campaigns involving clinical pharmacology units and RELIS in Trondheim and Tromsø visiting more than 700 GPs have focused on safe prescribing of NSAIDs and avoidance of antibiotic resistance, respectively. The RELIS network involves several working groups and an editor of social media (Facebook and Twitter). It also includes an IT consultant who maintains internet resources and develops apps for smartphones and bedside-tablet computers. The IT consultant provides statistics on the most accessed information in our home page.

RELIS is similar to other drug information centres in Europe with mainly patient-specific questions from physicians concerning drugs that affect the nervous system [2]. A crucial element is our collaborating national network with benefits of shared resources (Supplementary Fig. 1). Cooperation with our grant administrator (the Norwegian Medical Agency) through an annual plan provides RELIS the possibility to use funding from the Norwegian Ministry of Health and Care Services to develop beyond the traditional drug information centre. The network is now becoming more international as two Swedish drug information centres (Umeå and Gothenburg) have joined the RELIS database with publication of their answers.

## Electronic supplementary material

Supplementary figure 1 Benefits of a national network of drug information centres: RELIS (PDF 945 kb)
