# Supplementary material for: Benefits of a national network of drug information centres: RELIS
Source: Eur J Clin Pharmacol. 2016 Sep 15;73(1):125–6. doi: 10.1007/s00228-016-2129-7 (PMC5203813; doi:10.1007/s00228-016-2129-7)
Supplement: Supplementary file 1 — Benefits of a national network of drug information centres: RELIS (PDF 945 kb) [file 228_2016_2129_MOESM1_ESM.pdf]

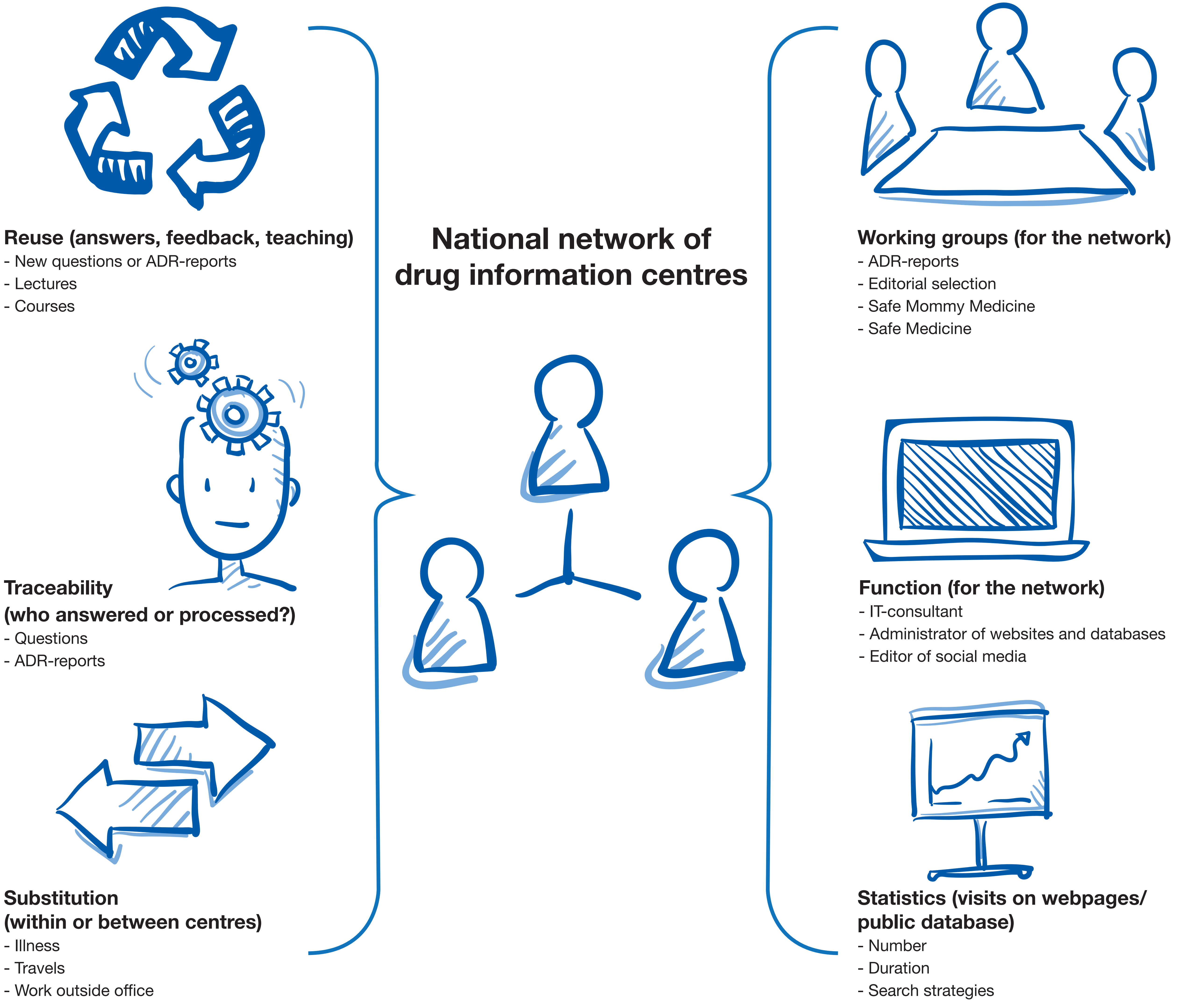
Reuse (answers, feedback, teaching)
- New questions or ADR-reports
- Lectures
- Courses
Traceability
(who answered or processed?)
- Questions
- ADR-reports
Substitution
(within or between centres)
- Illness
- Travels
- Work outside office
National network of
drug information centres
Working groups (for the network)
- ADR-reports
- Editorial selection
- Safe Mommy Medicine
- Safe Medicine
Function (for the network)
- IT-consultant
- Administrator of websites and databases
- Editor of social media
Statistics (visits on webpages/
public database)
- Number
- Duration
- Search strategies
